# Effects of Module Truncation of a New Alginate Lyase VxAly7C from Marine *Vibrio xiamenensis* QY104 on Biochemical Characteristics and Product Distribution

**DOI:** 10.3390/ijms23094795

**Published:** 2022-04-27

**Authors:** Luyao Tang, Mengmeng Bao, Ying Wang, Zheng Fu, Feng Han, Wengong Yu

**Affiliations:** 1 School of Medicine and Pharmacy, Ocean University of China, Qingdao 266003, China; tangluyao@stu.ouc.edu.cn (L.T.); baomengmengkk@gmail.com (M.B.); wangying998877@gmail.com (Y.W.); fuzheng@stu.ouc.edu.cn (Z.F.); 2 Laboratory for Marine Drugs and Bioproducts of Qingdao National Laboratory for Marine Science and Technology, Qingdao 266237, China; 3 Key Laboratory of Marine Drugs, Ministry of Education, Qingdao 266003, China; 4 Shandong Provincial Key Laboratory of Glycoscience and Glycoengineering, Qingdao 266003, China; 5 State Key Laboratory of Microbial Metabolism, School of Life Sciences and Biotechnology, Shanghai Jiao Tong University, Shanghai 200240, China

**Keywords:** carbohydrate-binding module, alginate lyase, brown algae, catalytic efficiency, product distribution

## Abstract

Alginate lyase has received extensive attention as an important tool for oligosaccharide preparation, pharmaceutical production, and energy biotransformation. Noncatalytic module carbohydrate-binding modules (CBM) have a major impact on the function of alginate lyases. Although the effects of two different families of CBMs on enzyme characteristics have been reported, the effect of two combined CBM32s on enzyme function has not been elucidated. Herein, we cloned and expressed a new multimodular alginate lyase, VxAly7C, from *Vibrio* *xiamenensis* QY104, consisting of two CBM32s at N-terminus and a polysaccharide lyase family 7 (PL7) at C-terminus. To explore the function of CBM32s in VxAly7C, full-length (VxAly7C-FL) and two truncated mutants, VxAly7C-TM1 (with the first CBM32 deleted) and VxAly7C-TM2 (with both CBM32s deleted), were characterized. The catalytic efficiency of recombinant VxAly7C-TM2 was 1.82 and 4.25 times higher than that of VxAly7C-TM1 and VxAly7C-FL, respectively, indicating that CBM32s had an antagonistic effect. However, CBM32s improved the temperature stability, the adaptability in an alkaline environment, and the preference for polyG. Moreover, CBM32s contributed to the production of tri- and tetrasaccharides, significantly affecting the end-product distribution. This study advances the understanding of module function and provides a reference for broader enzymatic applications and further enzymatic improvement and assembly.

## 1. Introduction

As the main structural polysaccharide of brown algae, alginate accounts for 22% to 44% of its dry weight [1]. It is an acidic heteropolysaccharide consisting of β-D-mannuronate (M) and α-L-guluronate (G), arranged in three kinds of blocks: homopolymeric M blocks (polyM), homopolymeric G blocks (polyG), and heteropolymeric blocks composed of alternating M and G (polyMG) [2]. Alginate is an important auxiliary material in the fields of medicine, food, agriculture, and energy [3,4,5]. As functional oligosaccharides, alginate oligosaccharides (AOS) have unique bioactivities and health benefits. The bioactivities of AOS are closely related to the degree of polymerization (DP) [6,7]. The 1,4 glycosidic bonds of alginate are cleaved by alginate lyases through a β-elimination reaction, and an unsaturated double bond between C4 and C5 is formed [8]. Alginate lyases have been used in the production of AOS, for protoplast production in brown algae, for prevention and treatment of bacterial biofilms, and in research on alginate structure and composition [9,10]. In past studies, thousands of alginate lyases have been found in marine microorganisms, brown seaweeds, and mollusks [11] and classified into PL families 5, 6, 7, 14, 15, 17, 18, 31, 32, 34, 36, 39, and 41 in the CAZy database [12]. Thus far, structures for 30 alginate lyases have been solved and are divided into four-fold classes: the β-jelly roll class (PL7, -14, -18, and -36), the (α/α)_n_ toroid class (PL5), the parallel β-helix class (PL6, -31), and the (α/α)_6_ toroid + anti-parallel β-sheet class (PL15, -17 and -39). Among the 12 structures of PL7 alginate lyases, only AlyB from *Vibrio splendidus* OU02 showed a full-length structure with a carbohydrate-binding modules family 32 (CBM32) and the PL7 catalytic domain connected by a noncanonical alpha helix linker [13].

Among the increasing number of alginate lyases that have been discovered, some alginate lyases are multimodule proteins containing the catalytic module and one or more CBMs [8,14,15]. Initially, these noncatalytic domains were found to bind crystalline cellulose as their primary ligand [16] and were defined as cellulose-binding domains. Subsequently, more CBMs binding diverse ligands have been identified [12]. To date, many CBMs have been identified experimentally or putatively. According to their amino acid sequence similarity, they are classified into 89 families in the CAZy database. CBMs do not have the catalytic ability but can affect various properties of the enzyme, such as targeting of substrates [13], disruption of insoluble substrate structures [17], or regulation of activity [18]. CBM_4_9, CBM13, CBM16, and CBM32 are the most common CBMs in alginate lyases. CBM32 is one of the most diverse CBM families [19], with a great depth of amino acid sequence variability and binding specificity. CBM32 was found in some GHs and PLs [20,21,22], and was involved in the recognition of substrates and changing the substrate specificity of enzymes such as chitosanase, mannanase, and N-acetylglucosaminidase [23,24,25]. CBM32 also enhanced enzyme activity by increasing affinity for substrates [26,27,28] and impacted the temperature stability [29].

At present, compared with the effect of a single CBM on the function of alginate lyase [13,15,30,31], the effects of two CBMs have been less studied. Several studies on two different families of CBMs, (e.g., CBM16-CBM32 and CBM35-CBM32) have found that different families of CBMs exert different functions for enzymatic characteristics, such as temperature stability, pH adaptability, and enzymatic activity [18,20,29]. However, the effects of the two combined CBM32s on enzyme function have not been reported. In this study, a new alginate lyase, VxAly7C, was cloned and expressed from *V. xiamenensis* QY104. Sequence analysis revealed that VxAly7C has three domains: two N-terminal CBM32 domains and a C-terminal alginate lyase catalytic module. We demonstrated the function of two CBM32s in VxAly7C by comparing the biochemical characteristics, modes of action, end product distributions, and catalytic efficiency of recombinant VxAly7C and its truncated mutants. This study promotes the understanding of CBM32, expands the application of CBM32 in the alginate lyase family, and provides rich material evidence for the combination and application of multimodule enzymes.

## 2. Results

### 2.1. Cloning and Sequence Analysis of the Alginate Lyase Gene

**Figure 1:**
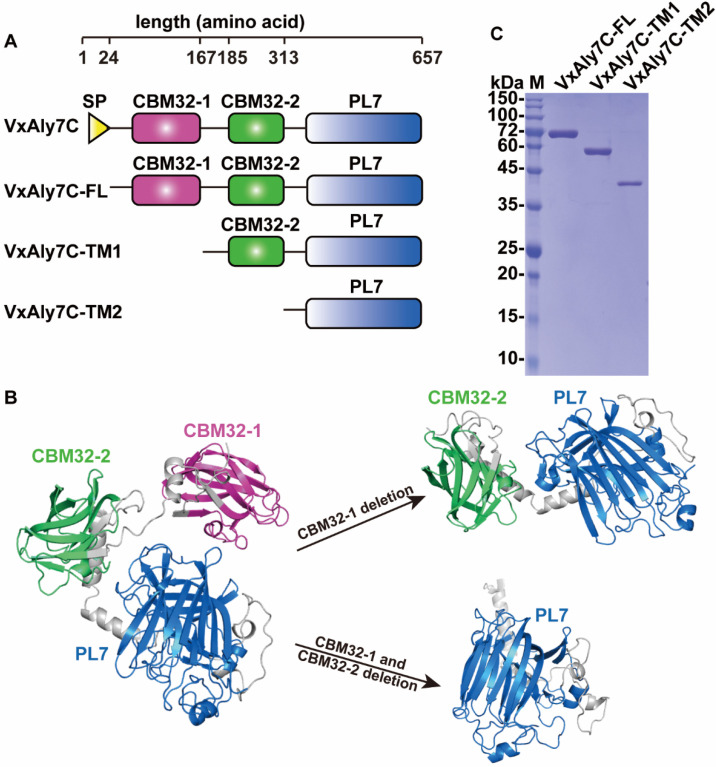
Construction of VxAly7C-FL, VxAly7C-TM1, and VxAly7C-TM2. (**A**), Domain structure of full-length VxAly7C, VxAly7C-TM1, and VxAly7C-TM2. (**B**), Models of VxAly7C-FL, VxAly7C-TM1, and VxAly7C-TM2. (**C**), Purified recombinant proteins were resolved by SDS-PAGE. Lane M, molecular weight markers.

**Figure 2:**
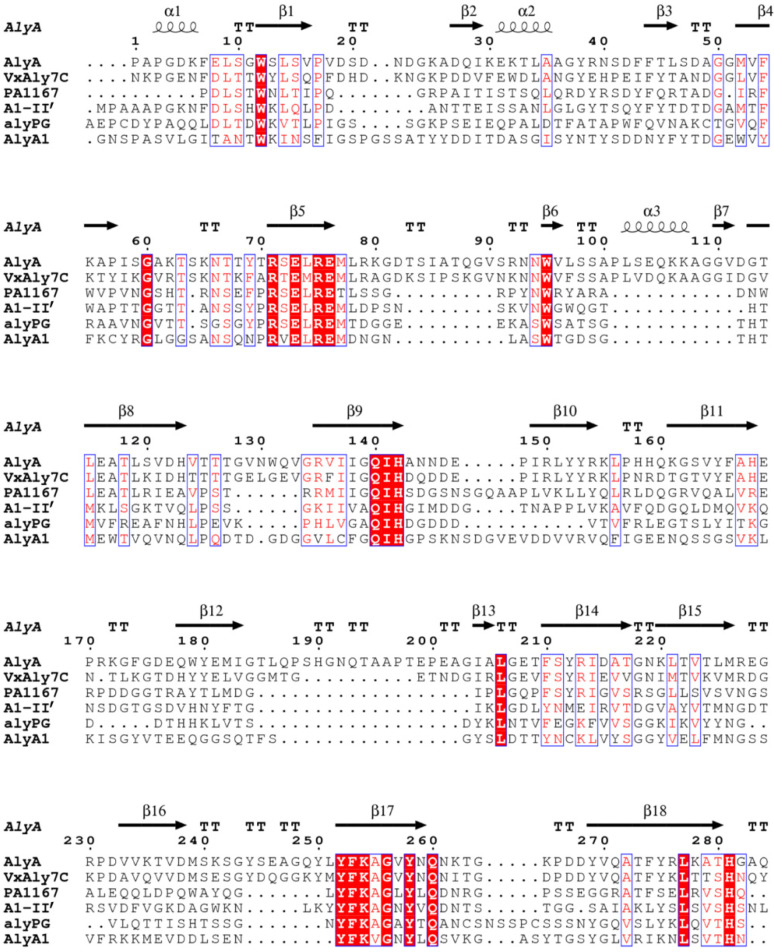
Multiple amino acid sequence alignment of VxAly7C with some crystallized PL7 enzymes. The secondary structure elements shown above are referenced according to AlyA. AlyA from *Klebsiella pneumoniae* subsp. *aerogenes* (AAA25049); PA1167, from *Pseudomonas aeruginosa* PAO1 (AAG04556); A1-II’, from *Sphingomonas* sp. A1 (BAD16656); AlyA1, from *Zobellia galactanivorans* DsiJT (CAZ95239); alyPG, from *Corynebacterium* sp. ALY-1 (BAA83339).

The alginate lyase gene *vxaly7c* consists of an ORF of 1974 bp, with a GC content of 41%, and encodes 657 amino acid residues. The SignalP 5.1 analyses indicate that the signal peptide of VxAly7C contains 24 amino acid residues (Met^1^-Ala^24^). Analyses using the NCBI conserved domain (CD) database, SMART, and Robetta indicate that VxAly7C contains three functional modules (Leu^25^-Lys^657^), including two CBM32s at the N-terminus (Val^44^-Phe^160^ named CBM32-1 and Val^186^-Phe^302^ named CBM32-2) and an alginate_lyase2 domain (Asp^391^-His^653^), which exhibits three highly conserved regions of the PL7 family: R(T/S/C/V)EL(G/R)(E/Q), YFKAGXYXQ, and Q(I/V)H (Figure 1A,B and Figure 2). Compared with the characterized PL7 alginate lyases listed in the CAZy database, VxAly7C has the highest identity (64.57%) with AlyPI from *Pseudoalteromonas* sp. CY24. The molecular weight and pI of the full-length enzyme (VxAly7C-FL), the first truncated mutant (VxAly7C-TM1, deletion of CBM32-1), and the other truncated mutant (VxAly7C-TM2, deletion of CBM32-1 and CBM32-2) deduced from their amino acid sequences are 70.73, 54.99, 38.67 Da and 5.26, 5.29, 5.38, respectively.

### 2.2. Heterologous Expression and Purification of Recombinant VxAly7C-FL, VxAly7C-TM1, and VxAly7C-TM2

The full-length enzyme VxAly7C-FL and its truncated mutants VxAly7C-TM1 and VxAly7C-TM2 were expressed in the *Escherichia coli* BL21 (DE3)/pET-24a (+) system. The recombinant VxAly7C-FL, VxAly7C-TM1, and VxAly7C-TM2 were purified and migrated as a single band of approximately 72, 56, and 40 kDa on the SDS-PAGE, respectively (Figure 1C), which were in good agreement with the calculated molecular weights of the recombinant protein fused with the (His)_6_ tag. The total protein yields from 1 L of the culture medium of recombinant VxAly7C-FL, VxAly7C-TM1, and VxAly7C-TM2 were 5.00 mg, 6.30 mg, and 5.20 mg, respectively. The specific activity of purified VxAly7C-FL was 40.41 U/nmol, which is lower than that of purified VxAly7C-TM1 and VxAly7C-TM2, 53.73 U/nmol and 54.62 U/nmol, respectively (Table 1). The above results indicate that the two CBM32s had an antagonistic effect on the catalytic domain, which was not conducive to the degradation of alginate by the enzyme.

To examine the binding ability of recombinant VxAly7C-FL, VxAly7C-TM1, and VxAly7C-TM2 to the substrate, we determined the enzymatic kinetic parameters. Compared with recombinant VxAly7C-FL, the *K*_m_ and *k*_cat_/*K*_m_ of recombinant VxAly7C-TM1 and VxAly7C-TM2 increased by 0.35- and 1.16-fold and by 1.35- and 3.25-fold, respectively (Table 2). The results showed that with the decrease in the CBM32s, both *K*_m_ and *k*_cat_/*K*_m_ had an upward trend, which was mainly due to the large increase in the *k*_cat_ value. It indicated that although CBM32s were helpful for the binding of enzymes to substrates, they were not conducive to the degradation of substrates by enzymes.

### 2.3. Biochemical Characteristics of Recombinant VxAly7C-FL, VxAly7C-TM1, and VxAly7C-TM2

**Figure 3:**
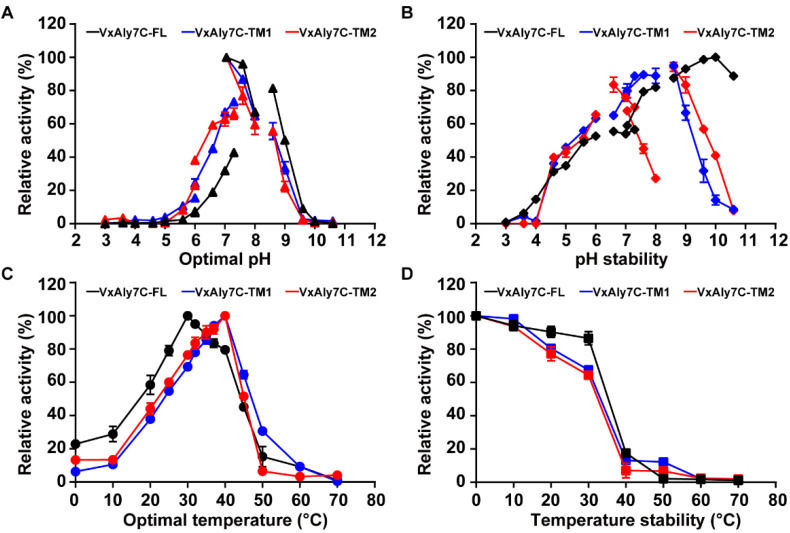
Effects of pH and temperature on recombinant VxAly7C-FL and its truncated mutants. Optimal pH (**A**), pH stability (**B**), optimal temperature (**C**), and temperature stability (**D**) of recombinant VxAly7C-FL and its truncated mutants.

The optimal pH was determined by measuring the enzymatic activities of recombinant VxAly7C-FL, VxAly7C-TM1, and VxAly7C-TM2 in different pH buffers. The results showed that the optimum pH of the three recombinant proteins occurred in Tris-HCl buffer, pH 7.05. Additionally, they maintained high activity at pH 7.0–9.0 (Figure 3A). The pH stability was determined by measuring the residual activity of recombinant proteins after 6 h of incubation in different pH buffers. Recombinant VxAly7C-FL, VxAly7C-TM1, and VxAly7C-TM2 exhibited the best pH stability in glycine-NaOH buffer at pH 10.0, 8.6, and 8.6, respectively. The recombinant VxAly7C-FL maintained more than 80% of the enzyme activity after incubation at pH 7.6–10.6 for 6 h. Recombinant VxAly7C-TM1 retained more than 80% of the enzyme activity after incubation at pH 7.6–9.0 for 6 h but less than 60% of the enzyme activity when the pH was higher than 9.0. However, when recombinant VxAly7C-TM2 was incubated under alkaline conditions for 6 h, less than 50% of the enzyme activity remained, especially when the pH was greater than 9.6, and only 40% of the enzyme activity remained (Figure 3B). The above results indicated that CBM32s, especially CBM32-2, were particularly important for the pH stability of VxAly7C in an alkaline environment.

The enzyme activities at different temperatures showed that the optimal temperatures of recombinant VxAly7C-FL, VxAly7C-TM1, and VxAly7C-TM2 were 30 °C, 40 °C, and 40 °C, respectively (Figure 3C), which indicated that compared with CBM32-2, CBM32-1 had a more significant effect on the optimum reaction temperature. Temperature stability was characterized by measuring the residual activity of recombinant proteins after incubation at different temperatures for 1 h. The results showed that recombinant VxAly7C-FL, VxAly7C-TM1, and VxAly7C-TM2 retained more than 80% of the enzymatic activity after incubation at 20 °C for 1 h. After incubation at 30 °C for 1 h, recombinant VxAly7C-FL retained 82% of the enzyme activity, and recombinant VxAly7C-TM1 and VxAly7C-TM2 retained 69% and 64% of the enzyme activity, respectively. After incubation at 40 °C for 1 h, the recombinant VxAly7C-FL, VxAly7C-TM1, and VxAly7C-TM2 had less than 20% enzyme activity (Figure 3D), which demonstrated that CBM32 could improve the temperature stability of VxAly7C, and the effect of CBM32-1 was more obvious.

### 2.4. Effects of NaCl, Metal Ions, Chelators, and Detergents on Alginate Lyase Activity

**Figure 4:**
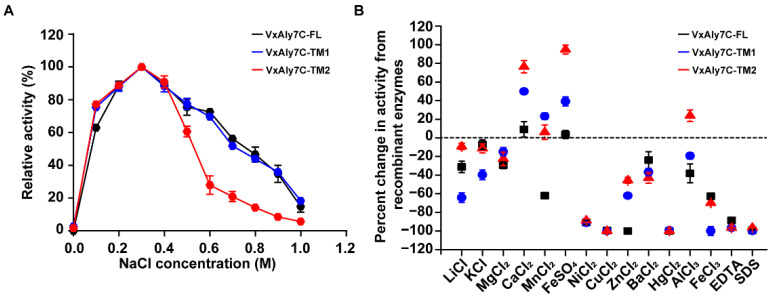
Effects of NaCl, metal ions, chelators, and detergents on recombinant VxAly7C-FL and its truncated mutants. Effect of the NaCl concentration (**A**) and metal ions, chelators, and detergents (**B**) on recombinant VxAly7C-FL, VxAly7C-TM1, and VxAly7C-TM2 activity.

NaCl is essential for the activity of recombinant VxAly7C-FL and its mutants since little enzymatic activity was detected in the absence of NaCl (Figure 4A). The highest activity was observed in the presence of 0.3 M NaCl. Recombinant VxAly7C-FL and VxAly7C-TM1 had similar adaptations to NaCl. The enzymatic activity of recombinant VxAly7C-TM2 decreased more than that of recombinant VxAly7C-FL and VxAly7C-TM1 with increasing NaCl concentration, suggesting that CBM32-2 might be more sensitive to changes in NaCl than CBM32-1. The effects of other metal ions, chelators, and detergents on the activities of recombinant VxAly7C-FL and mutants were shown in Figure 4B. The activities of recombinant VxAly7C-FL, VxAly7C-TM1, and VxAly7C-TM2 were strongly inhibited by 1 mM EDTA, SDS, Ni^2+^, Cu^2+^, or Hg^2+^. Zn^2+^ inactivated recombinant VxAly7C-FL, although recombinant VxAly7C-TM1 and VxAly7C-TM2 retained 37.5% and 54.4% activity, respectively. Compared with recombinant VxAly7C-FL, the activities of recombinant VxAly7C-TM1 and VxAly7C-TM2 were more significantly affected by Ca^2+^ and Fe^2+^. The presence of Ca^2+^ and Fe^2+^ increased the activity of recombinant VxAly7C-TM1 to 149.9% and 176.4%, respectively, and the activity of recombinant VxAly7C-TM2 to 139.1% and 194.8%, respectively. The presence of Mn^2+^ inhibited the activity of recombinant VxAly7C-FL but increased the activity of its truncation mutants. These results suggested that CBM32 altered the sensitivity of alginate lyase to certain metal ions.

### 2.5. Substrate Specificity of Recombinant VxAly7C and Its Truncated Mutants

**Figure 5:**
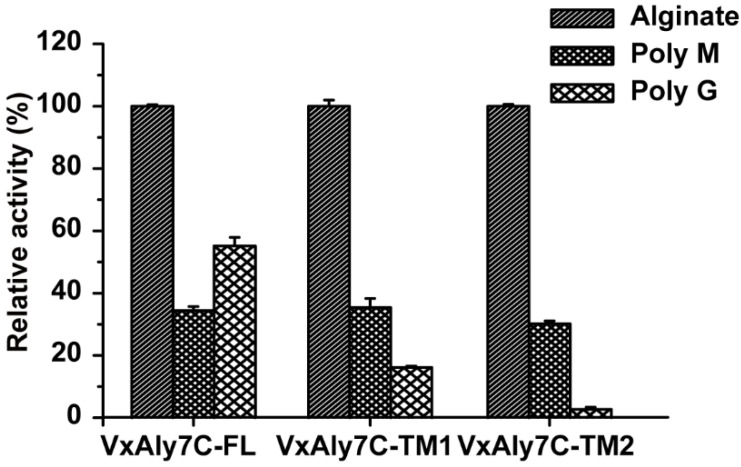
Substrate specificity of recombinant VxAly7C-FL and its truncated mutants toward alginate, polyM, and polyG.

Compared with the alginate substrate, the recombinant VxAly7C-FL, VxAly7C-TM1 and VxAly7C-TM2 had a similar degradation ability to polyM, which was approximately 30% of that of the alginate substrate. However, when polyG was used as a substrate, the degradation capacity of the recombinant proteins varied greatly. The abilities of recombinant VxAly7C-FL, VxAly7C-TM1, and VxAly7C-TM2 to degrade polyG were 55.2%, 16.1%, and 2.7% of that of alginate, respectively (Figure 5). Recombinant VxAly7C-FL favored the G-block over the substrate of the M-block, whereas recombinant VxAly7C-TM1 and VxAly7C-TM2 favored the M-block. The results suggested that CBM32 could enhance the recognition of G-block substrates by VxAly7C, thereby affecting the substrate preference of the enzyme.

### 2.6. Analysis of the Mode of Action and End Product Distribution

**Figure 6:**
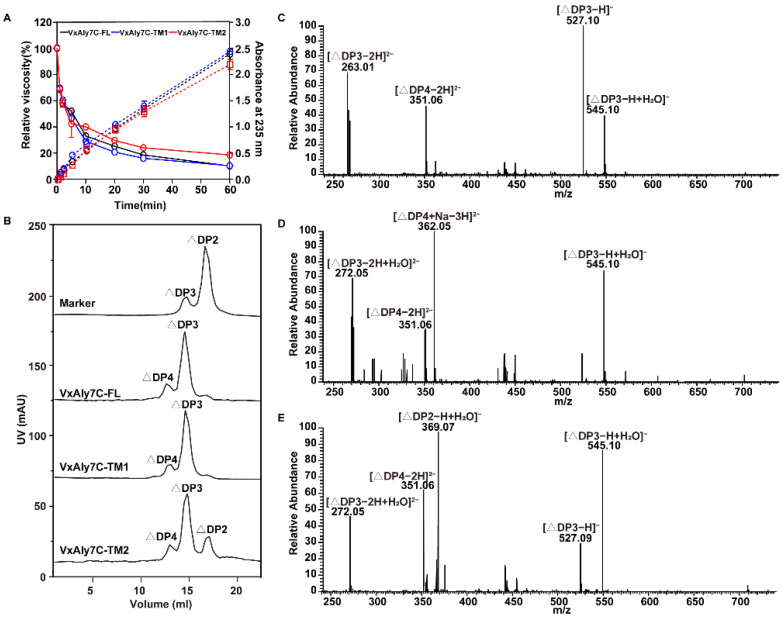
Modes of action and end products of alginate degradation by recombinant VxAly7C-FL and its truncated mutants. (**A**), Modes of action of recombinant VxAly7C-FL, VxAly7C-TM1, and VxAly7C-TM2. One mL of enzyme (10 U) was added to 9 mL of substrate solution, followed by incubation at 30 °C for different times. The changes in reduction in viscosity (solid line) and absorbance at 235 nm (dotted line) were measured. (**B**–**E**), End products of recombinant VxAly7C-FL, VxAly7C-TM1, and VxAly7C-TM2 analyzed by gel filtration chromatography and negative ion ESI-MS. Elution volume was 17.10 mL for unsaturated disaccharides (ΔDP2), 14.90 mL for unsaturated trisaccharides (ΔDP3), and 12.90 mL for unsaturated tetrasaccharides (ΔDP4).

The mode of action of recombinant VxAly7C-FL, VxAly7C-TM1, and VxAly7C-TM2 was examined using high-viscosity alginate as a substrate. The viscosity of a single spot was measured, and the reduction was calculated after incubation at 30 °C for 0, 1, 2, 5, 10, 20, 30, and 60 min with recombinant VxAly7C-FL, VxAly7C-TM1, and VxAly7C-TM2 (Figure 6A). The results showed a rapid drop in viscosity during the first 10 min and a slow drop during the last 50 min. Meanwhile, the absorbances at 235 nm recorded at different time points showed a steadily increasing trend (Figure 6A), suggesting that they acted as endo-type alginate lyases.

The end products of recombinant proteins were analyzed by gel filtration chromatography and negative ion ESI-MS. The results showed that the end degradation products of recombinant VxAly7C-FL and VxAly7C-TM1 were tri- and tetrasaccharides, and the peak area analysis showed that their molar ratios were 3.71:1.00 and 4.68:1.00, respectively (Figure 6B–D). The end degradation products of recombinant VxAly7C-TM2 were di-, tri-, and tetrasaccharides with a molar ratio of 1.71:4.00:1.00 (Figure 6B,E). The results suggested that CBM32 contributed to the production of tri- and tetrasaccharides and that CBM32-2 was less conducive to the production of disaccharides with low degrees of polymerization than CBM32-1. The above results demonstrated that CBM32s did not affect the mode of action of VxAly7C but significantly affected the distribution of degradation end products.

## 3. Discussion

With the in-depth exploration of marine polysaccharide-degrading enzymes, in addition to alginate lyases with only catalytic domains, many multimodule alginate lyases have emerged. However, most multimodule alginate lyases include one auxiliary catalytic module, such as CBM, and one catalytic domain [14,19], and only a few alginate lyases contain two or more auxiliary modules. The auxiliary catalytic module of the alginate lyase AlyQ from *Persicobacter* sp. CCB-QB2 includes CBM16-CBM32 [32], DP0100 from *Defluviitalea phaphyphila* Alg1 is the CBM35-CBM32-FN3 domain [20], and TsAly7B from *Thalassomonas* sp. LD5 contains CBM9-CBM32 [18]. In this study, we discovered the new alginate lyase VxAly7C with two CBM32s and explored the effects of the CBM32s on the catalytic activity, biochemical characteristics, and end product distribution by analysis of the full-length protein and its truncated mutants.

Compared with recombinant VxAly7C-FL, the catalytic activity of the mutants increased with the reduction of modules. The *k*_cat_/*K*_m_ of recombinant VxAly7C-TM1 with truncated CBM32-1 was 2.35 times that of VxAly7C-FL. The *k*_cat_/*K*_m_ of recombinant VxAly7C-TM2 containing only the catalytic domain was 4.25 times that of VxAly7C-FL and 1.82 times that of VxAly7C-TM1 (Table 2), indicating that CBM32s had an inhibitory effect on the enzymatic activity of VxAly7C. In our previous studies on the alginate lyase VxAly7B of *V. xiamenensis* QY104 [14], we found that CBM32 was not conducive to the catalysis of soluble substrates by the enzyme. The same phenomenon also occurred in alginate lyase AlyM from *Microbulbifer* sp. CGMCC 14061 [29] and AlyH from *Marinimicrobium* sp. H1 [33]. Although the *K*_m_ of recombinant VxAly7C-TM2 was higher than that of VxAly7C-FL and VxAly7C-TM1, the change in *k*_cat_ was more significant, increasing the *k*_cat_/*K*_m_ of recombinant VxAly7C-TM2. The enzymatic kinetic parameters of AlyQ from *Persicobacter* sp. CCB-QB2 also showed a similar trend, but its CBM32 contributes to the catalysis of the substrate by the enzyme. The *K*_m_, *k*_cat,_ and *k*_cat_/*K*_m_ of the truncated AlyQ mutants all showed a decreasing trend with the reduction of the domains in AlyQ [32]. In addition to AlyQ, the CBM32 of alginate lyase TsAly7B from *Thalassomonas* sp. LD5 [18] and glycoside hydrolases SsGalNagA from *Streptococcus suis* 05ZYH33 [21] both promoted enzyme activity. Differences in the sequence and structure of CBM32 might affect the flexibility of the full-length protein or the catalytic domain, thereby conferring different variations of enzymatic activity characteristics [34].

Similar to TsAly7B [18], CBM32s in VxAly7C increase the adaptability of the enzyme in an alkaline environment. Under alkaline conditions, as the amount of CBM32 increased, the pH stability of the recombinant protein improved (Figure 3B). The pH adaptability of surface charges to thermolysin-like proteases was investigated by inserting or removing charges on the protein surface [35]. Different CBMs caused pullulanase to show different degrees of activity at different pH values, suggesting that the effect of CBM on the pH adaptability of recombinant pullulanase might be due to the change in surface charge and the different binding ability to the substrate [36]. Therefore, the charge distribution of the protein caused by CBM32s in an alkaline environment might help maintain the normal conformation of VxAly7C. The presence of CBM32 has an important effect on the adaptability of the enzyme to temperature, and the roles played by different alginate lyases are not the same. CBM32 in AlyH [33] decreased the temperature stability of the enzyme, while CBM32 in Aly5 [37] increased the temperature stability of the enzyme. Recombinant VxAly7C-TM1 and recombinant VxAly7C-TM2 were not significantly different in optimum temperature and temperature stability but were significantly different from those of recombinant VxAly7C-FL. The optimum temperature of the truncated mutants changed from 30 °C to 40 °C for the full-length protein, and the temperature stability of the mutants decreased compared with that of recombinant VxAly7C-FL (Figure 3C,D), indicating that CBM32-1 has a greater impact on the temperature adaptability of the enzyme, while CBM32-2 has no significant effect. Models for VxAly7C-FL, VxAly7C-TM1, and VxAly7C-TM2 were generated using Robetta, and protein interactions were calculated by PIC (http://pic.mbu.iisc.ernet.in/ (accessed on 25 February 2022)). Compared with VxAly7C-TM1 and VxAly7C-TM2, we found that the presence of CBM32-1 increased one intraprotein hydrophobic interaction and two intraprotein main chain-side chain hydrogen bonds between CBM32-2 and the PL7 catalytic domain, which might increase the structural stability of VxAly7C-FL. CBM32s might be beneficial for maintaining rigid protein structure, as structural changes associated with increased protein stability are often described as leading to an increase in overall structural stiffness, which is likely to be accompanied by a decrease in catalytic performance [38,39].

As a substrate-binding domain, CBM has been receiving increased attention in substrate recognition research. At present, various types of CBMs are known to exhibit different mechanisms of action on substrate binding. CBM13 from the alginate lyase AlyL2 in *Agarivorans* sp. L11 predisposed the enzyme to degrade M-block substrates and increased the disaccharide content of the product [31]. The minimal degradation substrate of the CBM32 truncation mutant of Aly5 in *Flammeovirga* sp. MY04 changed from UDP5 to UDP6. The content of UDP2 and UDP3 in the product significantly decreased, while the UDP4-7 content significantly increased [37], indicating that the presence of CBM32 promoted enzyme binding and degradation of lower molecular weight oligosaccharides. The absence of N-terminal CBM32 resulted in a complex distribution of degradation products of Aly7A in *Vibrio* sp. W13 and was not conducive to the production of trisaccharides [40]. This shows that CBM32 causes Aly7A to adopt a unique mode of action on the substrate, positioning the cleavage of a trisaccharide. CBM32 in VxAly7C displayed a clear preference for substrate recognition. Both CBM32s contribute to the recognition and degradation of polyG by VxAly7C (Figure 5). The increase in the number of CBM32 was positively correlated to the content of the tetrasaccharide in the end product. The end product of recombinant VxAly7C-TM2 contained 25% disaccharides (Figure 6B), indicating that CBM32 contributed to the production of larger molecular weight oligosaccharide products.

**Figure 7:**
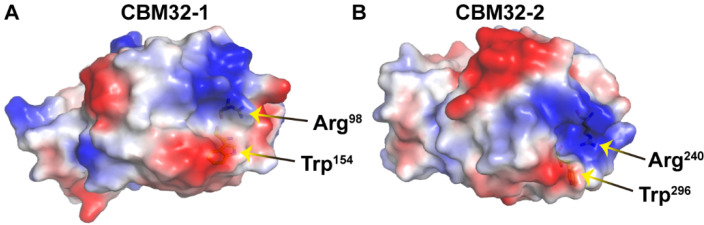
Electrostatic surface potential of CBM32-1 (**A**) and CBM32-2 (**B**) were generated by PyMOL.

The presence of substrate-binding residues in CBM32 also provides insights into the preferential binding of substrates and the specific distribution of products. Substrate binding experiments and crystal structure analysis demonstrated that CBM32 of AlyQ from *Persicobacter* sp. CCB-QB2 only bonded to the unsaturated sugar unit of the substrate, which might be a result of the high variability of the region around Trp^303^ that leads to the involvement of CBM32 in binding different substrates [32]. The loss of fluorescence polarization change for W129A of CBM32 in AlyB from *V. splendidus* OU02 toward the trisaccharide indicated a significant role of Trp^129^ in substrate binding [13]. Among the crystallized CBM32s, the CBM32-1 and CBM32-2 showed the highest identity (51.18% and 66.18%) with that of CBM32 from AlyB. The structures of CBM32s in VxAly7C were obtained by homologous modeling with SWISS-MODEL using CBM32 from AlyB (PDB ID: 5zu6). The results of homology modeling were consistent with the structure simulated by Robetta. Among them, Trp^154^ in CBM32-1, Trp^296^ in CBM32-2, Trp^303^ in AlyQ, and Trp^129^ in AlyB were in the same spatial position, suggesting that the conservation of Trp may contribute to the critical role of CBM32 in VxAly7C in substrate binding and product distribution. Furthermore, Arg^98^ in CBM32-1, Arg^240^ in CBM32-2, and Arg^248^ in AlyQ [32] correspond to steric positions, which also seem to be potential functional residues with interactions with the carboxyl group in the substrate. We represented the positions of Arg and Trp in CBM32s by electrostatic surface potential. The results showed that Arg was located in the alkaline-rich region, while Trp and Arg were spatially adjacent, suggesting that they were likely to be potential residues for interaction with substrates (Figure 7). Therefore, the potential substrate-binding residues in CBM32s might allow for it to act as a “controller” to regulate the specific recognition and degradation of substrates by VxAly7C.

## 4. Materials and Methods

### 4.1. Bacterial Strains, Plasmids, and Chemicals

*E. coli* strains DH5α and BL21(DE3) were cultured at 37 °C in Luria-Bertani (LB) broth or on LB agar supplemented with kanamycin sulfate (30 μg/mL) when relevant. Vector pET-24a (+) plasmids (Takara Co., Ltd., Dalian, China) were used for recombinant protein expression. The culture of *V. xiamenensis* QY104 and genome acquisition were as reported previously [14]. The source of the substrate was as described in previous reports [41]. All chemical reagents were of analytical grade.

### 4.2. Sequence Analysis

For functional annotation of the predicted proteins, identity analysis of amino acid sequences was performed using the BLAST algorithm on the NCBI (http://www.ncbi.nlm.nih.gov (accessed on 12 February 2022)). The theoretical isoelectric point (pI) and molecular weight (*M*_W_) were calculated using the Compute pI/*M*_W_ Tool (http://web.expasy.org/compute.pi/ (accessed on 20 December 2021)). Signal peptides were identified using the SignalP 5.1 server. Protein modules and domains were identified by using the SMART (http://smart.embl-heidelberg.de/ (accessed on 1 March 2016)) and NCBI conserved domain (CD) databases. The amino acid sequence alignment between VxAly7C and crystallized PL7 alginate lyases was obtained using ClustalW and further aligned with ESPript 3.0 (http://espript.ibcp.fr/ESPript/cgi-bin/ESPript.cgi (accessed on 5 January 2022)). Models for VxAly7C-FL, VxAly7C-TM1, and VxAly7C-TM2 were generated using Robetta (https://robetta.bakerlab.org/ (accessed on 25 February 2022)) and visualized by PyMOL. The sequence of VxAly7C was submitted to GenBank under Accession number OM793717.

### 4.3. Construction of Recombinant VxAly7C and Its Truncated Mutants

To express the full-length (without signal peptides) VxAly7C (VxAly7C-FL) and its truncated mutants VxAly7C-TM1 and VxAly7C-TM2, the appropriate DNA fragments were amplified by PrimeSTAR™ HS DNA Polymerase (Takara Co., Ltd., Dalian, China) using genomic DNA from *V. xiamenensis* QY104 as a template. The primers (PVxAly7C-FL-F/R, PVxAly7C-TM1-F, and PVxAly7C-TM2-F) are shown in Table S1. The PCR products were digested with NdeI and SalI and ligated into pET-24a (+).

### 4.4. Heterologous Expression and Purification of Recombinant Alginate Lyase

The constructs were transformed into *E. coli* BL21 (DE3) cells and initially cultured in LB broth at 37 °C and 160 r/min. The cells were grown until the OD_600_ reached 0.4-0.6, and the broth was supplemented with isopropyl-1-thio-β-D-galactoside (IPTG) at a final concentration of 0.1 mM to initiate protein expression before culturing at 18 °C and 160 r/min for an additional 24 h. The cells were harvested at 4 °C and 12,000 r/min for 10 min. The pellet was resuspended with binding buffer [20 mM phosphate buffer (PB), 500 mM NaCl] and crushed by a high-pressure crusher (JNBIO, Guangzhou, China). The crude enzyme solution was obtained by centrifugation at 4 °C and 12,000 r/min for 20 min. Recombinant VxAly7C-FL, VxAly7C-TM1, and VxAly7C-TM2 were purified from the soluble fraction using a HisTrap HP column (GE Healthcare, Stamford, CT, USA). The purity and molecular weights of the purified proteins were determined by SDS-PAGE on a 12.5% (*w*/*v*) resolving gel.

### 4.5. Enzyme Activity and Kinetic Parameter Assay

The alginate lyase activity was measured as the increase in absorbance at 235 nm. Unless otherwise stated, 100 μL of solution with 12 μg enzyme was reacted with 900 μL of substrate solution [0.3% (*w*/*v*) alginate, 20 mM PB plus 0.3 mM NaCl, pH 7.3] at 30 °C (recombinant VxAly7C-FL) or 40 °C (recombinant VxAly7C-TM1 and VxAly7C-TM2) for 10 min. One unit (U) was defined as the amount of enzyme required to increase the absorbance at 235 nm by 0.1 per min. For the studies of substrate specificity, polyM or polyG was used as the substrate. The kinetic parameters of recombinant VxAly7C-FL, VxAly7C-TM1, and VxAly7C-TM2 were measured by using 10 different alginate concentrations [ranging from 0.1% (*w*/*v*) to 1% (*w*/*v*)] of substrate solution at 30 °C or 40 °C for 3 min. The *K*_m_ and catalytic efficiency (*k*_cat_/*K*_m_) values were determined as previously reported [14].

### 4.6. Biochemical Characteristics of Recombinant VxAly7C-FL and Its Truncated Mutants

The optimal pH of the enzymatic activity was determined using 50 mM Na_2_HPO_4_-citric acid buffer (pH 3.0–6.0), 50 mM Na_2_HPO_4_-NaH_2_PO_4_ buffer (pH 6.0–8.0), 50 mM Tris-HCl buffer (7.0–8.0) and 50 mM glycine-NaOH buffer (pH 8.6–10.6) in the assay system. To determine the pH stability, the residual activity was measured after the enzymes were incubated in the above buffers for 6 h at 4 °C. The optimal temperature of the recombinant VxAly7C-FL and its truncated mutants was determined by measuring the activity at different temperatures (0, 10, 20, 25, 30, 32, 35, 37, 40, 45, 50, 60, 70 °C). The temperature stability of enzymes was examined by measuring the residual activity after the enzymes were incubated at 0–70 °C for 1 h in 20 mM PB (pH 7.3). The effects of NaCl on recombinant VxAly7C-FL, VxAly7C-TM1, and VxAly7C-TM2 activity were examined by monitoring the enzymatic activity at various concentrations (0–1 M) of NaCl in substrate solution [0.3% (*w*/*v*) alginate, pH 7.3]. The effects of metal ions, chelators, and detergents on recombinant VxAly7C-FL, VxAly7C-TM1, and VxAly7C-TM2 activity were investigated by measuring the enzymatic activity in the presence of various cations or chelators at a final concentration of 1 mM.

### 4.7. Substrate Specificity, Mode of Action, and End Product Distribution Analysis

To study the substrate specificity, alginate, polyM, and polyG (0.3% (*w*/*v*) in 20 mM PB plus 300 mM NaCl, pH 7.3) were used as substrates in the standard enzymatic assay described above. One mL (10 U) of purified enzyme was added to 9 mL of high-viscosity alginate substrate solution and incubated at 30 °C for 0, 1, 2, 5, 10, 20, 30, or 60 min. After boiling for 10 min to halt the reaction, the viscosity and UV absorbance at 235 nm of each reaction mixture were measured to determine the mode of action of recombinant proteins.

The reaction mixture containing 0.1 mL (10 U) purified enzymes and 0.4 mL substrate solution was incubated at 30 °C overnight. The end products were analyzed using a Superdex peptide 10/300 GL gel filtration column (GE Healthcare, Madison, WI, USA) equilibrated with 0.2 M NH_4_HCO_3_ and detected at 235 nm by fast protein liquid chromatography (FPLC). The end products were analyzed by negative ion electrospray ionization mass spectrometry (ESI-MS) from 0 to 1500 m/z. The scope with no significant product peaks is not shown.

## 5. Conclusions

In this study, we cloned and expressed a new multimodule alginate lyase, VxAly7C, consisting of two CBM32s at the N-terminus and a PL7 domain at the C-terminus. The function of CBM32s in VxAly7C was elucidated by the characterization of full-length (VxAly7C-FL) and two truncated mutants, VxAly7C-TM1, and VxAly7C-TM2. The *k*_cat_/*K*_m_ of recombinant VxAly7C-TM2 was 829.95 ± 9.77, which was 1.82 and 4.25 times that of recombinant VxAly7C-TM1 and VxAly7C-FL, respectively, indicating that CBM32s attenuated the enzyme activity. The stability of recombinant VxAly7C-FL in an alkaline environment (pH 7.6–10.6) was better than that of the truncated mutants, and it retained 82% of the enzyme activity at 30 °C for 1 h, which was higher than the 69% or 64% enzyme activity of the truncated mutants. Furthermore, the presence of CBM32s predisposed recombinant VxAly7C to preferentially degrade G-block substrates and increased the content of tri- and tetrasaccharides in the end product. This work would enhance the understanding of the function of the domains within alginate lyases and provide new insights for multimodule utilization.

## Figures and Tables

**Table 1:** The specific activity of recombinant VxAly7C-FL, VxAly7C-TM1, and VxAly7C-TM2.

Protein	Specific Activity (U/mg)	Molecular Weight (kDa)	Specific Activity (U/nmol)
VxAly7C-FL	557.82	72.47	40.41
VxAly7C-TM1	946.34	56.72	53.73
VxAly7C-TM2	1351.47	40.41	54.62

**Table 2:** Enzyme kinetic parameters of recombinant VxAly7C-FL and its truncated mutants.

	VxAly7C-FL	VxAly7C-TM1	VxAly7C-TM2
*K*_m_ (mM)	8.91 ± 0.11	12.09 ± 0.07	19.23 ± 0.16
*k*_cat_ (s^−1^)	1734.86 ± 10.49	5523.81 ± 12.31	15,960 ± 5.14
*k*_cat_/*K*_m_ (s^−1^·mM^−1^)	194.93 ± 2.25	456.89 ± 2.45	829.95 ± 9.77

## Data Availability

The sequence of VxAly7C was submitted to GenBank under Accession number OM793717.

## Supplementary Materials

The following supporting information can be downloaded at: https://www.mdpi.com/article/10.3390/ijms23094795/s1.
