# Supplementary material for: Effects of Module Truncation of a New Alginate Lyase VxAly7C from Marine Vibrio xiamenensis QY104 on Biochemical Characteristics and Product Distribution
Source: Int J Mol Sci. 2022 Apr 27;23(9):4795. doi: 10.3390/ijms23094795 (PMC9102848; doi:10.3390/ijms23094795)
Supplement: Supplementary file 1 [file ijms-23-04795-s001.zip › ijms-1663999-supplementary.pdf]

Supplementary material

Effects of module truncation of a new alginate lyase VxAly7C from marine *Vibrio xiamenensis* QY104 on biochemical characteristics and product distribution

Luyao Tang[1,2,3,4,5], Mengmeng Bao[1,2,3,4], Ying Wang[1,2,3,4], Zheng Fu[1,2,3,4], Feng Han[1,2,3,4]*, Wengong Yu[1,2,3,4]*

Table S1. PCR primers for the recombinant VxAly7C and its truncated mutants

Primer name	Primer sequence (5'-3')	Recombinant enzyme
PVxAly7C-FL-F	GGAATTC**CATATG**CAGAGTGG TTGTGTGCAAAGT	VxAly7C-FL
PVxAly7C-FL-R	ACGC**GTCGAC**TTTATATTGATT ATGTGATGTTGTAA	VxAly7C-FL, VxAly7C-TM1, VxAly7C-TM2
PVxAly7C-TM1-F	GGAATTC**CATATG**GACGCGTG CTCTAATCAAGAA	VxAly7C-TM1
PVxAly7C-TM2-F	GGAATTC**CATATG**GATAACCA TATTATTACTGATAAG	VxAly7C-TM2

The bases in bold are recognition sites for restriction endonuclease enzymes.
